# Differences of PPARD Expression in the Liver of Cattle with Different Marbling Grades

**DOI:** 10.3390/ani16132096

**Published:** 2026-07-06

**Authors:** Kaiyou Wang, Qi Wang, Qinyu Wang, Shuaiying Tian, Ying Qi, Lin Zhang, Baokui Xing, Tuliguer, Qiuling Li

**Affiliations:** 1 Hebei Key Laboratory of Animal Diversity, Langfang Key Laboratory of Cell Engineering and Application, College of Life Sciences, Langfang Normal University, Langfang 065000, China; wangkaiyou0226@163.com (K.W.); 18103149870@163.com (Q.W.); 18232877282@163.com (Q.W.); 18617911396@163.com (S.T.); qiying_doc@163.com (Y.Q.); zhanglin@lfnu.edu.cn (L.Z.); 2 Ordos Caojin Livestock Breeding Company, Ordos 017000, China; baobao0714@sohu.com (B.X.); tuliguerzhao@163.com (T.)

**Keywords:** *PPARD*, lipid deposition, marbling, fatty acid transport, lipid droplets

## Abstract

Fat deposition determines beef marbling grade and meat quality, but the underlying molecular mechanisms remain unclear. This study aimed to investigate the role of bovine PPARD in lipid deposition, especially its effect on the expression of fatty acid transport genes (*CD36*, *FATP1*, and *FABP1*) and the lipid droplet-associated gene (*PLIN2*) in liver tissues from cattle with different marbling grades. The mRNA abundance and protein levels in liver tissues from thirty-one Wagyu × Angus crossbred beef cattle (25–26 months old) with different marbling grades (according to GB/T 29392-2022, based on the marbling richness of the longissimus dorsi muscle at the 12th–13th rib interface) were analyzed by RT-qPCR and Western blot, respectively. Additionally, *PPARD* was knocked down and overexpressed in bovine mammary epithelial cells to validate its effects on lipid-metabolism-related genes. The results showed that the mRNA levels of *CD36*, *FATP1*, *PPARD*, *RXRA*, *RXRB*, and *PLIN2* were significantly higher (*p* < 0.01) in livers tissues from the A3 and A4 groups (high marbling) than in those from the A1 and A2 groups (low to moderate marbling). Western blot analysis revealed significantly higher PPARD protein expression in the A3 and A4 groups (high marbling) than that in the A1 and A2 groups (low to moderate marbling) (*p* < 0.05). It should be noted that the sample size of Group A4 is only 2, and the results of this group should be considered as a preliminary trend that needs to be validated with larger sample sizes. Cellular experiments confirmed that *PPARD* knockdown significantly decreased mRNA expressions of *FABP1*, *CD36*, and *PLIN2* (*p* < 0.01), while *PPARD* overexpression significantly increased their mRNA levels (*p* < 0.05). These results indicate a positive correlation between PPARD expression and the transcriptional levels of genes involved in fatty acid transport and lipid droplet storage, suggesting that PPARD may be associated with hepatic lipid metabolism and potentially contribute to marbling development. These findings suggest that the PPARD signaling pathway contributes to hepatic lipid deposition and may play a role in marbling formation in beef cattle.

## Simple Summary

The palatability and commercial value of beef are strongly influenced by the amount and distribution of intramuscular fat, whose typical deposition pattern is known as marbling. However, the molecular mechanisms driving this process in cattle remain poorly understood. This study investigated the role of a nuclear receptor, peroxisome proliferator-activated receptor delta (PPARD), in regulating hepatic lipid metabolism and its potential association with marbling formation. By analyzing liver tissues from beef cattle with different marbling scores and performing cellular experiments in bovine mammary epithelial cells, we found that animals with higher marbling grades exhibited elevated expression of *PPARD*, along with increased levels of genes responsible for fatty acid transport (*CD36*, *FATP1*) and lipid droplet formation (*PLIN2*). Activating PPARD in cell models increased mRNA expressions of these genes, whereas inhibiting PPARD reduced their expressions. These results indicate that PPARD may facilitate lipid deposition in the liver by promoting both fatty acid uptake and lipid droplet assembly. Given the liver’s central role in systemic lipid metabolism, this PPARD-mediated pathway may influence the availability of circulating fatty acids, thereby contributing to intramuscular fat deposition and marbling development. Elucidating this mechanism provides a scientific foundation for genetic selection and breeding strategies aimed at improving beef marbling and overall meat quality, benefiting both producers and consumers.

## 1. Introduction

Intramuscular fat content has an important impact on beef quality and has a positive impact on meat flavor. Marbling refers to white spots or strips of intramuscular fat between muscle fiber bundles. Marbling is the typical form of intramuscular fat deposition and serves as a core indicator for evaluating the eating quality and commercial value of beef. The formation of marbling is a dynamic process driven by the activation of preadipocytes, the uptake of fatty acids, and the accumulation of lipid droplets in muscle and controlled by systemic lipid metabolism and local regulatory networks. It arises from the complex interaction among genetic, nutritional, and environmental factors and involves the multi gene regulation mechanism that has not been fully defined [1].

Lipid droplets are the primary organelles for lipid storage in eukaryotic cells. Perilipin 2 (PLIN2), a key structural protein on the lipid droplet surface, stabilizes lipid droplets by escaping ubiquitin-proteasomal degradation and thereby modulates lipolysis and lipid storage [2]. While *PLIN2* function has been characterized in adipocytes of humans and model animals, its specific roles and post-transcriptional regulation in bovine lipid metabolism across tissues remain unclear.

Peroxisome proliferator activated receptor (PPAR) is a nuclear receptor that controls adipocyte plasticity and energy homeostasis [3]. *PPARD* subtype is the central regulator of fatty acid transport, oxidation, and mitochondrial function [4,5,6]. In rodents, *PPAR δ* and *PPAR α* synergistically regulate the transcription of *UCP1* and *PGC-1 α* in brown adipocytes to maintain the thermogenic process [7]. In addition, PPAR δ also improves insulin signaling in liver and muscle, regulates insulin sensitivity, and inhibits inflammation by promoting M2 polarization of adipose tissue macrophages [8,9]. In pig lipid metabolism, *PPARD* polymorphism is related to its lipid deposition [10]. Other studies have shown that *PPARD* can compensate for *PPAR α* deficiency to maintain lipid homeostasis in mouse skeletal muscle [11]. These results suggest that the *PPARD* gene may be a candidate gene for regulating the characteristics of bovine lipid deposition. *PPARD* is a key component of the PPAR signaling pathway and a major transcription factor involved in adipose tissue homeostasis and energy metabolism. However, the precise mechanisms by which it regulates downstream target gene networks through this pathway remain to be fully elucidated. Although the PPAR signaling pathway plays an important role in fat metabolism, the mechanism of fat deposition and meat quality changes in cattle, especially the mechanism of PPAR subtype specificity, which is still unclear.

The liver is an important organ involved in the development of intramuscular adipose tissue [12]. Intramuscular fat deposition may be driven by preferential shunting of hepatic lipid reserves to muscle, suggesting a “liver–muscle” regulatory network that mediates tissue crosstalk [13]. However, the relationship between hepatic mRNA expression levels of lipid metabolism-related genes and intramuscular fat deposition remains poorly understood in cattle.

Here, we hypothesize that *PPARD* promotes hepatic lipid deposition and may contribute to intramuscular fat (IMF) development by transcriptionally activating fatty acid transporters (*CD36*, *FATP1*, and *FABP1*) and the lipid droplet-associated gene *PLIN2*. To test this hypothesis, we first examined the mRNA expression of *PPARD* and its target genes in liver tissues from cattle with different marbling grades. To further validate the functional role of *PPARD* in regulating these genes, we used bovine mammary epithelial cells (BMECs) as an in vitro model. Although BMECs do not fully recapitulate hepatic or muscle physiology, they were selected for this study because they exhibit robust de novo lipogenesis, express key lipogenic transcription factors, and retain high transfection efficiency, making them a practical and reliable tool for gene overexpression and knockdown studies of lipid metabolism. We acknowledge that future validation using primary hepatocytes or hepatic cell lines would be valuable to confirm these findings in a liver-specific context. This study provides correlative evidence for the potential involvement of *PPARD* in the regulatory network of bovine lipid metabolism and offers insights into marbling formation.

## 2. Materials and Methods

### 2.1. Sample Collection

All 31 cattle used in this study were Wagyu × Angus crossbred females, sourced from the same farm and raised under identical feeding and management conditions. They were randomly selected from a single cohort of 25–26-month-old cattle with no more than two generations of full-sib or half-sib relationships, which minimizes potential pedigree and sire effects. However, we acknowledge that detailed pedigree structure and formal sire effect analysis were not available. The liver tissue samples were collected and immediately frozen in liquid nitrogen and subsequently stored at −80 °C until mRNA and protein extraction. The marbling was evaluated based on the national standard GB/T 29392-2022 [14]. The intramuscular fat distribution characteristics of the longissimus dorsi muscle between the 12th and 13th ribs of the beef carcass were marbling scored by professionals through visual inspection.

### 2.2. Cell Culture

Bovine mammary epithelial cells (BeNa Biotechnology, Beijing, China) were grown in DMEM/F12 medium (Gibco, Grand Island, NY, USA) supplemented with 10% fetal bovine serum (Gibco, Grand Island, NY, USA), 100 U/mL penicillin, and 100 mg/mL streptomycin (Solarbio, Beijing, China, catalog no. P1400). Cells were cultured in T-25 cell flasks in an incubator at 37 °C with 5% CO_2_ and were passaged when cell density reached 90% at a 1:3 ratio in a new flask.

### 2.3. Cell Transfection and Treatment

The pcDNA3.1 *PPARD* plasmid and *PPARD* siRNA had been developed by Shanghai Sangon Biotech (Shanghai, China), and the sequence is provided in Table 1. The transfection of plasmids of overexpression and siRNAs into the cell was performed using Lipofectamine 6000 transfection reagent (Beyotime Biotechnology, Shanghai, China, catalog no. C0526-0.5 mL). The blank plasmid or si-NC (Sangon Biotech, Shanghai, China) sequence was transfected in the control group. The cell density in the 6-well plates (Nest, Wuxi, China) was seeded 24 h before transfection by 1 × 10^6^ cells/well. As soon as the cell confluence was observed to be 60–70 percent, the basic medium (DMEM/F12, Gibco, Grand Island, NY, USA) was substituted one hour prior to transfection. It was 100 pmol of siRNA (or the negative control siRNA) or plasmid into 5 μL of Lipofectamine 6000 in 250 μL of basic medium. The medium was then changed to complete medium after 4–6 h of incubation. Cells were then incubated in a 37 °C with 5 percent CO_2_ (Thermo Fisher Scientific, Waltham, MA, USA) incubator over a period of 24–48 h, after which they were further analyzed.

### 2.4. Total RNA Extraction and Reverse Transcription

Total RNA was extracted from bovine liver tissues and bovine mammary epithelial cells using TRIzol reagent (Invitrogen, Carlsbad, CA, USA, catalog no. 15596026CN). Tissue samples were ground into powder in liquid nitrogen and then mixed with 1 mL of TRIzol, while cell samples were washed with PBS (Solarbio, Beijing, China) after removing the culture medium, followed by direct addition of 1 mL of TRIzol and scraping for lysis. After homogenization, 200 μL of chloroform was added to the samples, followed by vigorous shaking and centrifugation to separate phases. The upper aqueous phase was collected, and an equal volume of isopropanol was added to precipitate the RNA. The RNA pellet was washed twice with 75% ethanol, air-dried, and dissolved in 20 μL of DEPC-treated water. RNA concentration and purity (OD260/280 1.8-2.0) were measured using a microspectrophotometer. Reverse transcription was performed according to the manufacturer’s instructions of the reverse transcription kit (Thermo Fisher Scientific, Waltham, MA, USA, catalog no. 4368814), using a PCR instrument (Bio-Rad, Hercules, CA, USA) under the following conditions: 25 °C for 5 min, 42 °C for 60 min, and 70 °C for 5 min. The cDNA was stored at −20 °C for later use.

### 2.5. RT-qPCR

Real-time quantitative PCR (RT-qPCR) was performed using the SYBR Green (BIOSHARP, Beijing, China, catalog no. BL698A) method to determine the mRNA expression levels of target genes in liver tissues and bovine mammary epithelial cells. All reactions were carried out on a Bio-Rad IQ5 system (Bio-Rad, Hercules, CA, USA). The thermal cycling program was as follows: 95 °C for 5 min, followed by 40 cycles of 95 °C for 30 s and 60 °C for 30 s.

The housekeeping gene *β-actin* was used as an internal control. Relative gene expression was calculated using the 2^−ΔΔCt^ method. Each sample of livers or cell treatments was run in three technical replicates. The numbers of biological replicates of liver samples for different marbling grades (A1–A4) were as follows: A1 = 7, A2 = 12, A3 = 10, A4 = 2. The sample size reflects the natural distribution of marbling grades in the cattle population under study, with extreme high marbling (A4, grades 4) occurring less frequently than lower grades. No artificial selection or exclusion was applied; the numbers represent the actual availability within each grade category. However, we acknowledge that the number of the A4 group (*n* = 2) is very small, which limits statistical power and the precision of variance estimation.

Primers were designed using Primer Premier 5.0 software (Premier Biosoft, Palo Alto, CA, USA), with exon–exon junctions to avoid genomic DNA amplification. The sequences of primer and length of products are listed in Table 2. The RT-qPCR reaction mixture is detailed in Table 3.

The specificity of all primer pairs was confirmed by melting curve analysis, which yielded single peaks for all target genes. *β-actin* was used as the reference gene, with Ct values showing low variation across samples.

### 2.6. Western Blot

Proteins in tissues and cells were extracted using PMSF and lysis buffer and boiled for denaturation after adding loading buffer. Supernatants of cells were removed, and cells were washed once with PBS, followed by lysis in PMSF (Beyotime, Shanghai, China, catalog no. ST507) and cell lysis buffer for Western and IP (Beyotime, Shanghai, China, catalog no. P1103). Liver tissue samples were grinded and then underwent lysis. The supernatant was collected and centrifuged at 10,000r for 3 min. After removing cell debris, the obtained supernatant was combined in 2 × sample loading buffer. Proteins were separated by the SDS-PAGE electrophoresis. Following electrophoretic transfer of proteins onto PVDF membranes (Immobilon-P, Merck Millipore, Burlington, MA, USA), nonspecific binding was blocked overnight in 5% skimmed milk and washed three times with TBST. The membranes were incubated with primary antibodies against anti-PPARD (Beyotime, Shanghai, China, AF7800, 1:1000), anti-PLIN2 (Sangon, Shanghai, China, catalog no. D261248, 1:800), anti-β-actin (Solarbio, Beijing, China, catalog no. K101527P), and anti-GAPDH (Beyotime, Shanghai, China, catalog no. AF1186, 1:4000) at room temperature for 1 h. After the additional washing three times with TBS-T, the membranes were incubated with horseradish peroxidase conjugated secondary antibodies (Beyotime Biotechnology, Shanghai, China) at room temperature for 1 h. Subsequently, the membranes were washed again with TBST and treated with chemiluminescent substrate. Images were acquired using developer and analyzed with ImageJ (version 1.54g, National Institutes of Health, Bethesda, MD, USA).

### 2.7. Statistics

GraphPad Prism 9.5 (GraphPad Software, San Diego, CA, USA) was used for statistical analysis and graphing. Western blot band intensities were quantified using ImageJ, and the relative expression of target proteins was calculated after normalization to the internal control.

For each gene or protein analyzed individually, one-way ANOVA was performed with marbling grade (A1, A2, A3, A4) or treatment (interference, negative control, and blank control) as the fixed factor, followed by Tukey’s HSD post hoc test for multiple comparisons. Because the liver tissue groups were unbalanced (*n* = 7, 12, 10, and 2 for A1–A4, respectively), least-squares means (LSMeans) were estimated for group comparisons using the general linear model (GLM) procedure. Prior to ANOVA, normality of residuals and homogeneity of variances were verified using the Shapiro–Wilk and Levene’s tests, respectively; both assumptions were met for all analyzed variables. Specifically, one-way ANOVA was applied to (i) RT-qPCR results of fatty acid transport-related genes and nuclear receptor-related genes in liver tissues, (ii) RT-qPCR results of lipid metabolism-related genes in cell knockdown and overexpression models, and (iii) Western blot results of PPARD and PLIN2 proteins in liver tissues. For Western blot analysis of PPARD and PLIN2 proteins in cell knockdown and overexpression models, as well as for transfection efficiency validation, an unpaired *t*-test was used. All data are presented as mean ± SEM, and statistical significance was set at *p* < 0.05.

## 3. Results

### 3.1. RT-qPCR Detection of PPARD Signaling Pathway Lipid Deposition-Related Genes in Liver Tissues of Beef Cattle with Different Marbling Grades

**Figure 1:**
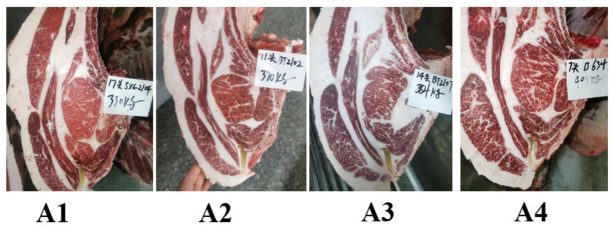
Representative image of different marbling grade-indicated intramuscular fat. Marbling is a key phenotypic indicator of beef lipid accumulation and an essential evaluation index for beef quality and commercial value. The four gradient marbling grades (A1–A4) shown in the figure represent gradual increases in intramuscular fat content and uniform fat distribution in bovine longissimus dorsi muscle. Specifically, A1 exhibits the least marbling (fewest fat flecks), A2 shows slightly more, A3 has relatively abundant marbling, and A4 displays the most abundant and uniform pattern. The distinct phenotypic differences among groups provide intuitive macroscopic evidence for graded lipid deposition in beef cattle and lay a phenotypic foundation for subsequent verification of the regulatory relationship between *PPARD* expression level and marbling formation as well as lipid metabolism. Note: Sample IDs and their corresponding body weights. The Chinese term “头” in the figure indicates “head” (i.e., animal number). The sample IDs SX6-2104, BT2102, BT2107, and B634 correspond to body weights of 330 kg, 370 kg, 364 kg, and 409 kg, respectively. A1–A4 indicate marbling grade groups.

Figure 1 illustrates the intramuscular fat deposition characteristics corresponding to four marble vein grades (A1–A4) in this study. RT-qPCR was employed to detect the mRNA expression levels of genes related to the *PPARD* signaling pathway and lipid metabolism in liver tissues of beef cattle across different grades.

**Figure 2:**
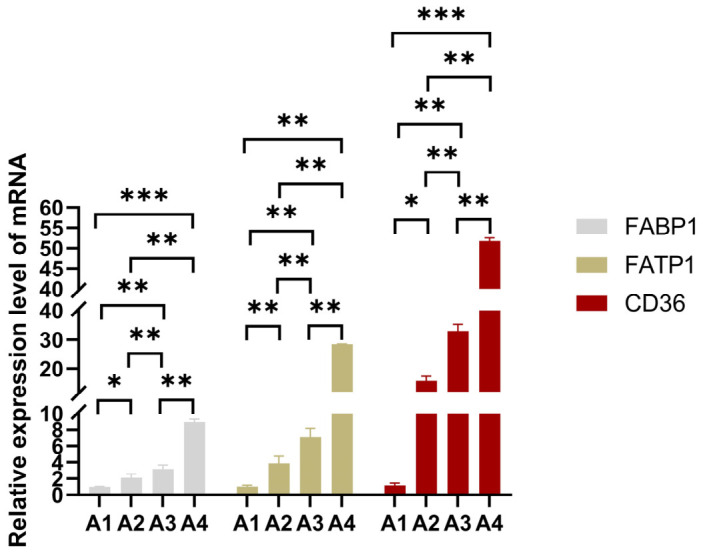
Relative mRNA expression levels of fatty acid transport-related genes (*FABP1*, *FATP1*, and *CD36*) in liver tissues of beef cattle with different marbling grades. Fatty acid transport is the initial and rate-limiting step of intracellular lipid deposition. *FATP1* and *CD36* serve as key transmembrane fatty acid transporters responsible for extracellular fatty acid uptake, while *FABP1* mediates intracellular fatty acid binding and trafficking to downstream lipid metabolic pathways. The significantly elevated mRNA expression of these three genes in high-marbling groups (A3, A4) indicates that enhanced fatty acid transport capacity is closely coupled with intramuscular fat deposition. These results suggest that the upregulation of fatty acid transport-related genes is an important molecular basis for the high marbling phenotype of beef cattle and provides preliminary evidence for the downstream regulatory mechanism of *PPARD* in lipid metabolism. Note: * indicates significance at the 0.05 level, ** indicates significance at the 0.01 level, *** indicates significance at the 0.001 level.

**Figure 3:**
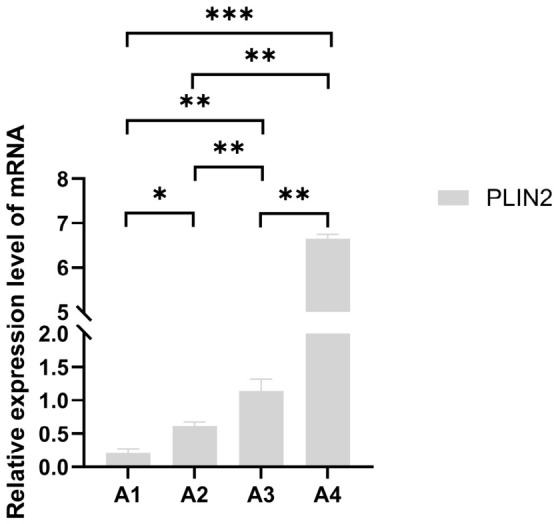
Relative mRNA expression level of the lipid droplet marker gene in liver tissues of beef cattle with different marbling grades. *PLIN2* is a core structural gene for lipid droplet formation, which functions to maintain lipid droplet stability, prevent intracellular lipolysis, and promote lipid storage accumulation, serving as a key terminal effector gene of lipid deposition. The gradually upregulated expression of *PLIN2* with the increase of marbling grade indicates that the enhanced transcription level of *PLIN2* accelerates lipid droplet formation and storage in bovine liver. This trend demonstrates that the activation of lipid droplet assembly is synchronized with the improvement of beef marbling quality, confirming that *PLIN2*-mediated lipid droplet deposition is closely associated with lipid metabolism characteristics in cattle. Note: * indicates significance at the 0.05 level, ** indicates significance at the 0.01 level, *** indicates significance at the 0.001 level.

**Figure 4:**
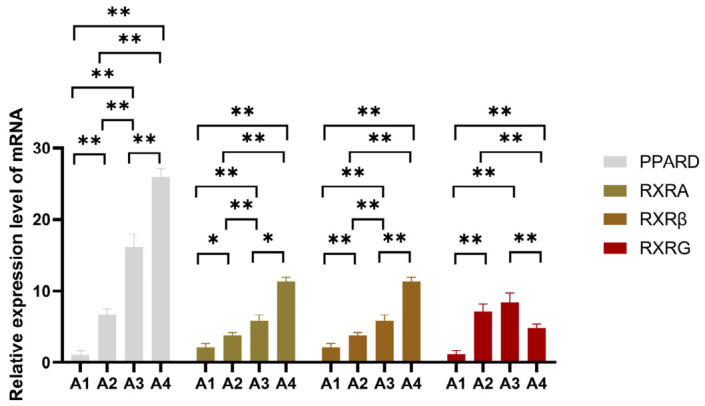
Relative mRNA expression levels of nuclear receptor-related genes (*PPARD*, *RXRA*, *RXRB*, and *RXRG*) in liver tissues of beef cattle with different marbling grades. Nuclear receptor genes are an essential component of the lipid metabolic signaling axis, which are closely involved in lipid synthesis and storage in livestock liver. The mRNA levels of *PPARD*, *RXRA*, and *RXRB* were significantly increased in high marbling groups, showing a consistent upward trend with the elevation of intramuscular fat grade, while *RXRG* expression exhibited no obvious differences among all groups. These results indicate that the differential expression of nuclear receptor subtypes is closely associated with bovine lipid deposition and marbling formation. The altered expression of *PPARD* and its main heterodimer partners (*RXRA*/*RXRB*) is highly synchronized with the improvement of beef marbling traits, providing phenotypic evidence for the correlation between nuclear receptor gene expression and lipid metabolism characteristics in cattle. Note: * indicates significance at the 0.05 level, ** indicates significance at the 0.01 level.

The results showed that the mRNA levels of fatty acid transport genes (*CD36*, *FATP1*), nuclear receptor genes (*PPARD*, *RXRA*, *RXRB*), and lipid droplet formation gene (*PLIN2*) were significantly upregulated with high marbling grade (*p* < 0.05); *FABP1* and *RXRG* showed significant differences only between group A1 and A4 (*p* < 0.05, Figure 2, Figure 3 and Figure 4). It should be noted that the sample size of group A4 (*n* = 2) is very limited. Although its mRNA level shows a further upward trend, the statistical power is low, and the reliability of the arithmetic mean estimates for this group is constrained by the small sample size. The statistical significance and biological robustness of these results therefore require verification in larger cohorts.

The above results indicate that genes in the *PPARD* signaling pathway that are linked in the metabolism of lipids have a significant effect on the process of lipid deposition in the liver with different marbling grade of beef cattle. This study only confirms the correlation between the two, without verifying a direct causal relationship. Changes in gene expression correlate with altered liver lipid metabolism and circulating fatty acid supply, which may indirectly provide a material basis for intramuscular fat deposition.

### 3.2. Protein Expression of PPARD and PLIN2 in Beef Cattle Liver Tissues with Various Marbling Grading

The Western blot bands were subjected to semi-quantitative analysis of grayscale values using ImageJ software. GAPDH and β-actin were used as an internal control for normalization of protein expression levels.

**Figure 5:**
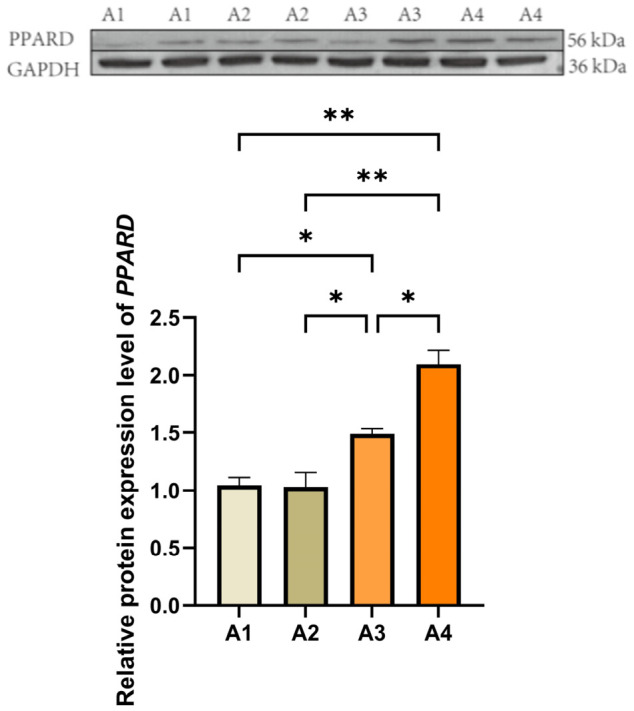
Protein expression levels of PPARD in liver tissues of beef cattle with different marbling grades. PPARD is a key nuclear receptor protein involved in hepatic lipid metabolism. The protein abundance of PPARD was gradually increased alongside the elevation of beef marbling grade, with significantly higher expression in the high-marbling A3 and A4 groups compared with the low-marbling A1 and A2 groups. The consistent increasing trend of PPARD protein level with marbling quality indicates that PPARD protein expression is positively correlated with intramuscular fat deposition capacity in beef cattle. These protein-level phenotypic data further support the close association between PPARD expression and bovine lipid accumulation and correspond to the variation trend of its mRNA expression in liver tissues. Note: * indicates significance at the 0.05 level, ** indicates significance at the 0.01 level.

The results indicated significant differences in PPARD protein expression in liver tissues of beef cattle with different marble vein grades (*p* < 0.01). Specifically, the expression level of PPARD protein in A3 grade beef cattle was significantly higher than that in A1 (*p* < 0.05) and A2 grades (*p* < 0.05); the expression level was the highest in A4 grade, significantly higher than that in A1 (*p* < 0.01), A2 (*p* < 0.01), and A3 grades (*p* < 0.05, Figure 5; original membranes in Supplementary Figure S1). However, the A4 group contained only two biological replicates; therefore, this highest expression value cannot be taken as a definitive conclusion but merely suggests an upward trend in PPARD protein expression with increasing marbling grade, which needs to be confirmed with a larger sample size. The expression of PPARD protein showed a positive correlation trend with marble vein grades, suggesting that the upregulation of PPARD expression in the liver may be associated with enhanced systemic lipid metabolic activity, providing a potential material basis for intramuscular fat deposition.

**Figure 6:**
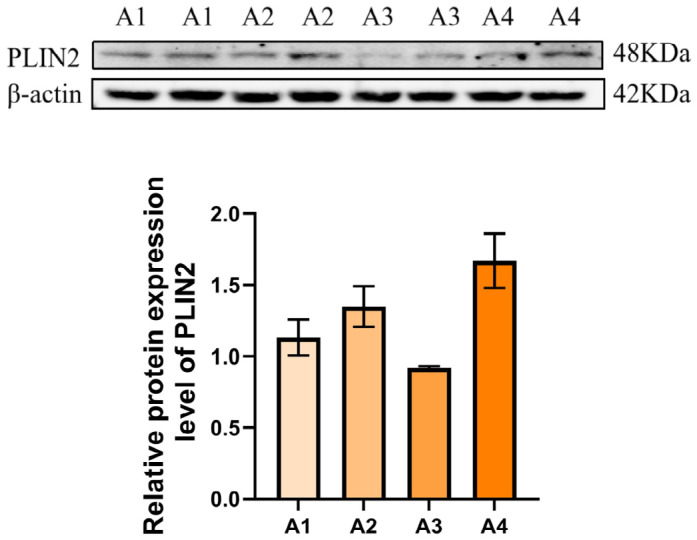
Protein expression levels of PLIN2 in liver tissues of beef cattle with different marbling grades. PLIN2 is a crucial structural protein of intracellular lipid droplets, which participates in maintaining lipid droplet stability and lipid storage homeostasis in hepatic tissues. In the present study, no significant differences in PLIN2 protein abundance were observed among beef cattle groups with different marbling grades. These results reveal that the PLIN2 protein level in bovine liver tissues is relatively stable during graded lipid deposition. This inconsistent trend between PLIN2 protein expression and its mRNA expression suggests that post-translational modification may be involved in maintaining the steady state of PLIN2 protein, which is closely associated with the homeostasis of hepatic lipid metabolism in cattle.

It is worth noting that there was no significant statistical difference in PLIN2 protein expression among the groups (*p* > 0.05, Figure 6; original membranes in Supplementary Figures S2 and S3). This is inconsistent with the aforementioned result that “*PLIN2* mRNA levels significantly increase with the increase in marbling grade”, suggesting that there may be post-transcriptional regulation mechanisms (such as differences in translation efficiency, protein degradation regulation, etc.) of the *PLIN2* gene in bovine liver tissue. However, this mechanism was not experimentally verified in this study, and its specific molecular processes remain to be further explored in the future.

### 3.3. Validation of PPARD Knockdown and Overexpression in Cell Models

**Figure 7:**
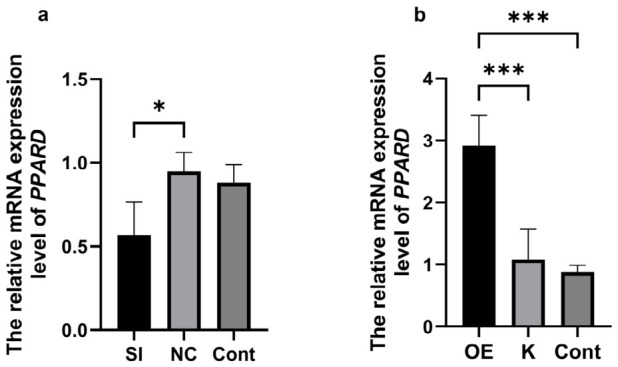
Validation of *PPARD* knockdown and overexpression in bovine mammary epithelial cells, supporting its role in regulating lipid deposition. (**a**) Transfection with *PPARD*-specific siRNA (SI) significantly reduced *PPARD* mRNA expression compared with negative control (NC) and blank control (Cont), confirming effective knockdown. (**b**) Transfection with *PPARD* overexpression vector (OE) markedly increased *PPARD* mRNA levels relative to empty vector (EV) and Cont, verifying successful overexpression. These results establish valid cell models for investigating *PPARD*-mediated regulation of fatty acid transport and lipid droplet formation. Data are mean ± SD; Note: * indicates significance at the 0.05 level, *** indicates significance at the 0.001 level.

**Figure 8:**
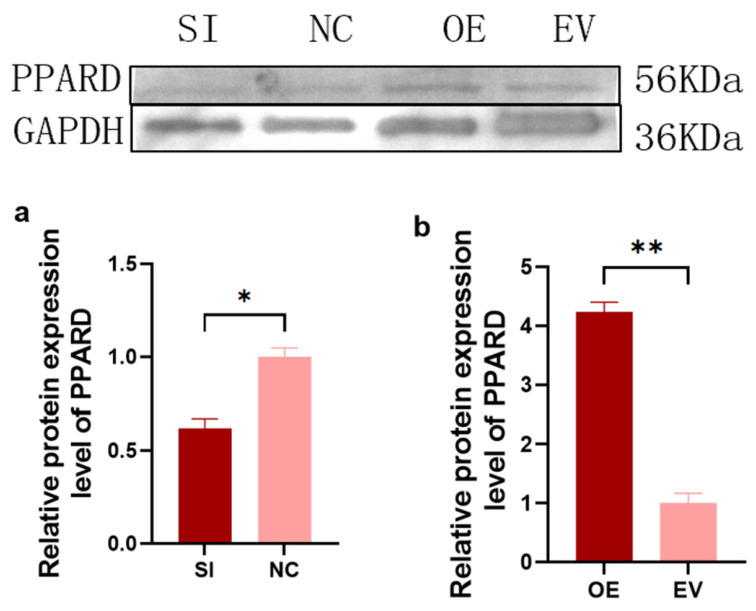
Validation of protein expression efficiency of *PPARD* knockdown and overexpression in bovine mammary epithelial cells. (**a**) *PPARD* interference effect: compared with the negative control group (NC), the *PPARD* specific siRNA interference group (SI) showed a significant decrease in the relative expression level of PPARD protein, confirming that siRNA can effectively silence endogenous PPARD protein expression in cells and successfully construct a *PPARD* knockdown cell model; (**b**) overexpression effect of *PPARD*: compared with the empty vector control group (EV), the *PPARD* overexpression plasmid transfection group (OE) showed a significant increase in the relative expression level of PPARD protein, indicating that overexpression vectors can efficiently upregulate PPARD protein and achieve gene overexpression. The above results demonstrate the successful construction of a *PPARD* knockdown and overexpression cell model, providing a reliable cell assay system for further exploration of *PPARD* regulation of lipid deposition and lipid metabolism-related functions and molecular mechanisms in bovine mammary epithelial cells. Note: * indicates significance at the 0.05 level, ** indicates significance at the 0.01 level.

RT-qPCR and Western blot were used to verify the effectiveness of the interference and overexpression model at the transcription and translation levels. The findings indicated that mRNA and the protein levels were significantly lower in the SI-*PPARD* group than those in the negative control (Nc) (Figure 7 and Figure 8, original membranes in Supplementary Figures S4 and S5) as well as significantly higher in the OE-*PPARD* group than that in the empty vector control (EV) and empty control (Cont). These results establish the positive establishment of *PPARD* knockdown and overexpression cell models.

### 3.4. RT-qPCR Detection of PPARD Signaling Pathway Lipid Deposition-Related Genes in Bovine Mammary Epithelial Cells

**Figure 9:**
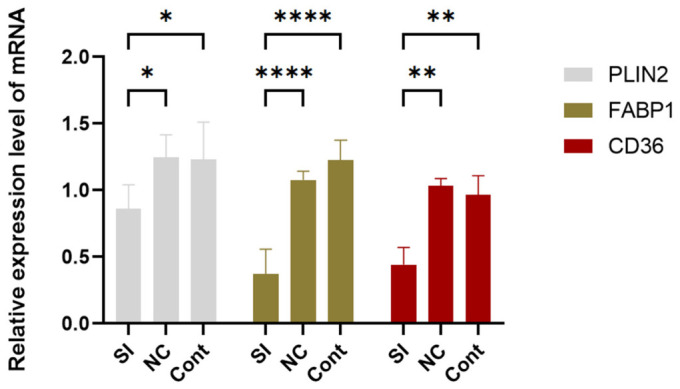
Relative mRNA expression of lipid metabolism-associated genes in bovine mammary epithelial cells with *PPARD* knockdown. After effective knockdown of *PPARD* expression, the mRNA levels of fatty acid transport-related genes (*FABP1*, *CD36*) and lipid droplet formation-related gene (*PLIN2*) were significantly decreased compared with the control groups. The downregulated expression of these lipid metabolism-associated genes is closely correlated with the decreased *PPARD* level. These cellular data indicate that low *PPARD* expression is associated with reduced transcription efficiency of fatty acid uptake and lipid storage genes, providing cellular-level evidence for the intrinsic association between *PPARD* expression and lipid metabolic characteristics in bovine mammary epithelial cells. Note: * indicates significance at the 0.05 level, ** indicates significance at the 0.01 level, **** indicates significance at the 0.0001 level.

**Figure 10:**
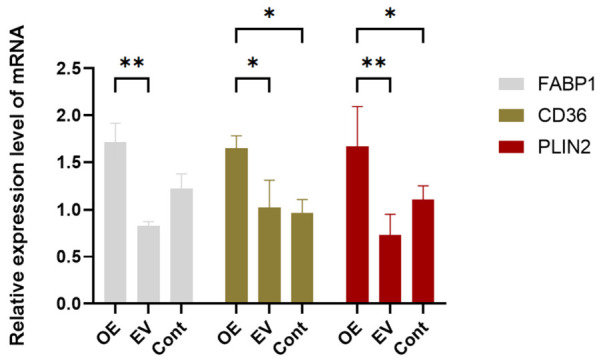
Relative mRNA expression of lipid metabolism-associated genes in bovine mammary epithelial cells with *PPARD* overexpression. After successful overexpression of *PPARD*, the mRNA levels of fatty acid transport genes (*FABP1*, *CD36*) and lipid droplet formation gene (*PLIN2*) were significantly increased relative to the blank and empty vector control groups. The upregulated transcription of these lipid metabolic genes is closely correlated with elevated *PPARD* expression. This cellular observation indicates that high *PPARD* expression is associated with increased transcriptional levels of genes related to fatty acid uptake and lipid droplet accumulation, which further confirms the intrinsic association between *PPARD* expression and lipid metabolic characteristics in bovine mammary epithelial cells. Note: * indicates significance at the 0.05 level, ** indicates significance at the 0.01 level.

We used real-time quantitative polymerase chain reaction (RT-qPCR) to detect the mRNA expression levels of lipid metabolism-related genes in bovine mammary epithelial cells after *PPARD* interference (SI-*PPARD*, Figure 9) and overexpression (OE-*PPARD*, Figure 10). The aim of this experiment was to verify the expression association between *PPARD* and lipid metabolism-related genes rather than simulating physiological processes in the liver or skeletal muscle.

The results showed that compared with the negative control group (NC) and the blank control group (Cont), there were significant differences in the mRNA expression levels of *FABP1*, *CD36*, and *PLIN2* in the SI-*PPARD* group (*p* < 0.01). *PPARD* interference significantly downregulated the expression of fatty acid transport genes *FABP1* and *CD36* (*p* < 0.01), and the mRNA level of lipid droplet structure-related gene *PLIN2* was also significantly reduced (*p* < 0.05).

On the other hand, compared with the empty vector control group (EV) and the blank control group (Cont), the mRNA expression levels of *FABP1*, *CD36*, and *PLIN2* were significantly upregulated in the OE-*PPARD* group (*p* < 0.05).

The above interference and overexpression experimental results were mutually confirmed and were consistent with the gene expression trend in liver tissue in vivo, indicating that the expression changes of *PPARD* were positively correlated with the transcriptional levels of *FABP1*, *CD36*, and *PLIN2*, suggesting that *PPARD* may be associated with the regulation of these lipid metabolism-related genes in bovine mammary epithelial cells, providing cytological evidence for further investigation of the role of *PPARD* in bovine lipid metabolism.

### 3.5. Western Blot Detection of PLIN2 Protein in Bovine Mammary Epithelial Cells

**Figure 11:**
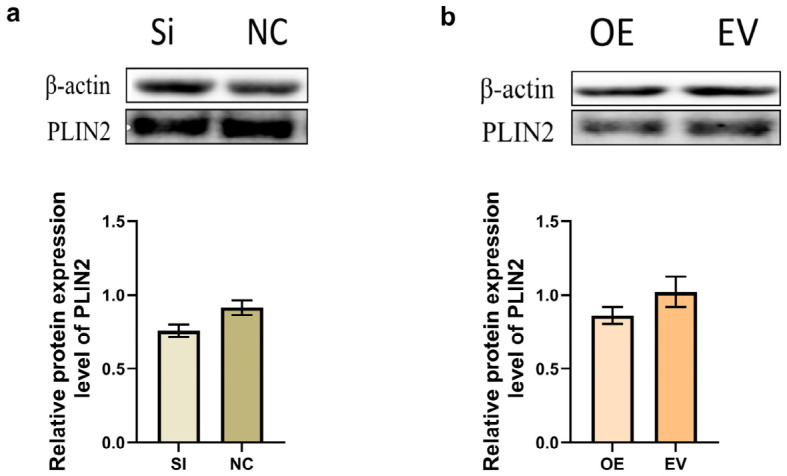
Relative protein expression of PLIN2 in bovine mammary epithelial cells with *PPARD* knockdown and overexpression. (**a**) PLIN2 protein expression in PPARD knockdown group compared to the negative control (NC) group. (**b**) PLIN2 protein expression in PPARD overexpression group compared to the empty vector (EV) control group. No significant differences in PLIN2 protein levels were observed among the knockdown, overexpression, and corresponding control groups. The stable protein abundance of PLIN2 under altered *PPARD* expression conditions indicates that PLIN2 protein translation or stability is not sensitive to changes in *PPARD* levels. This inconsistent trend between *PLIN2* protein expression and its mRNA expression is closely associated with possible post-translational modification and protein homeostasis maintenance in bovine mammary epithelial cells.

Western blot was used to detect the expression of *PLIN2* following *PPARD* knockdown and overexpression in bovine mammary epithelial cells. No significant differences were observed among the groups (Figure 11, original membranes in Supplementary Figures S6 and S7).

## 4. Discussion

This study preliminarily explored the correlation between peroxisome proliferator-activated receptor δ (*PPARD*) and lipid metabolism as well as intramuscular fat deposition in cattle by analyzing the expression differences of lipid metabolism-related genes in liver tissues of beef cattle with different marbling grades and combining with experiments on functional loss and gain in bovine mammary epithelial cells. The main findings are as follows: The mRNA and protein levels of PPARD in liver tissues were significantly positively correlated with marbling grades; the expression patterns of fatty acid transport genes *CD36*, *FATP1* and lipid droplet structure-related gene *PLIN2* were consistent with that of *PPARD*; in bovine mammary epithelial cells, knockdown of *PPARD* significantly downregulated the transcription levels of *FABP1*, *CD36*, and *PLIN2*, while overexpression significantly upregulated the transcription levels of these genes. It is worth noting that although the transcription level of *PLIN2* was affected by *PPARD*, no corresponding changes were observed in its protein level in both in vivo liver tissues and in vitro cell experiments.

It should be noted that the sample sizes among the marbling grade groups were uneven, with only 2 animals in the A4 group compared with 10 in the A3 group. This disparity reduces the statistical power for the A4 group and limits the robustness of any conclusions drawn from this highest marbling grade. Accordingly, the A4 data should be interpreted as exploratory observations reflecting tentative trends rather than definitive results. The primary evidence for high-grade fat deposition in this study comes from the A3 group (*n* = 10), which provides more reliable statistical support. The trends observed in the A4 group warrant further validation in future studies with larger sample sizes.

The above results suggest that *PPARD* positively regulates the transcription of genes related to fatty acid transport and lipid droplets in bovine liver tissues and mammary epithelial cells. The upregulation of its expression may be related to the material basis of enhanced systemic lipid metabolism and intramuscular fat deposition in beef cattle. The inconsistency between the transcription and translation levels of *PLIN2* indicates the presence of complex post-transcriptional regulatory mechanisms in the lipid metabolism process of cattle. Its specific function and molecular mechanism still require further investigation. This study provides basic data for understanding the molecular regulation of bovine lipid metabolism, but its direct regulatory effect on intramuscular fat deposition still needs to be verified in primary intramuscular preadipocytes.

### 4.1. PPARD Positively Regulates Fatty Acid Transmembrane Transport and Intracellular Trafficking

Membrane transporters are key regulators of lipid metabolism, and the “flip-flop” movement of fatty acids across the lipid bilayer is itself a rate-limiting step. Therefore, the transmembrane transport of fatty acids is critical for lipid metabolism [15,16]. *CD36* and *FATP1* are major membrane proteins that mediate the entry of long-chain fatty acid into cells [17,18], and their expression levels directly influence the cellular capacity for exogenous fatty acid uptake [19,20]. In this study, the mRNA levels of *CD36* and *FATP1* in the liver tissues of the A3 group (*n* = 10) were significantly higher than those in the A1 and A2 groups (*p* < 0.05) and were positively correlated with *PPARD* expression; the A4 group (*n* = 2) showed a further increasing trend in mRNA levels. It should be noted that, owing to the limited sample size of the A4 group, these data were not included in formal statistical comparisons and should be interpreted only as preliminary trend indications. In vitro bovine mammary epithelial cell experiments further confirmed that *PPARD* overexpression significantly upregulated *CD36* mRNA, while interference downregulated it, suggesting that *PPARD* may be involved in the transcriptional regulation of these transporter genes. This finding is consistent with previous research on the regulation of lipid uptake in rodents and pigs by the PPAR family [21,22,23]. When fatty acid supply increases, cells synthesize triglycerides and deposit them into existing lipid droplets, leading to droplet expansion [24]. Thus, *PPARD*-mediated regulation of transporter genes may represent an upstream initiation step in hepatic lipid deposition, which could indirectly influence intramuscular fat deposition. However, as the in vivo data are observational and the functional validation was conducted in bovine mammary epithelial cells rather than in hepatocytes, a direct causal relationship between *PPARD* expression and hepatic lipid deposition has not been established.

It is worth noting that, although the upregulation of fatty acid transport gene expression suggests an enhanced cellular fatty acid uptake capacity, this study did not assess liver triglyceride content or lipid droplet formation, thus it is unable to directly prove an increase in liver lipid deposition. Meanwhile, unlike the expression trends of *CD36* and *FATP1*, although the lipid droplet structure-related gene *PLIN2* increases at the mRNA level with the upregulation of *PPARD* expression, no corresponding changes were observed at the protein level in both in vivo liver tissues and in vitro cell experiments, suggesting that the post-transcriptional regulation mechanism of lipid metabolism in the bovine liver may play a significant role. Therefore, the upregulation of transporter gene expression mediated by *PPARD* can only reflect the enhanced activity of liver lipid metabolism, and whether it ultimately leads to net liver lipid accumulation still requires further verification. As the central hub of systemic lipid metabolism, the enhanced lipid metabolic activity in the liver may provide more circulating fatty acid substrates for intramuscular fat deposition, but the direct causal relationship between the two has not yet been established.

The expression of intracellular fatty acid-binding protein *FABP1* is also influenced by *PPARD*. FABP1 not only for directs internalized fatty acids to organelles such as the endoplasmic reticulum for esterification but also its own gene expression can be regulated by *PPARD* [25,26], thereby enhancing its transcriptional activity. Functional studies have shown that *FABP1* is involved not only in promoting lipid deposition but also in regulating fatty acids partitioning. *FABP1* knockout shifts fatty acids toward oxidative breakdown, thereby reducing lipid deposition in the liver [24]. In this study, *PPARD* interference significantly decreased *FABP1* mRNA levels, while overexpression increased them, suggesting a potential positive correlation between PPARD and FABP1 expression, which might involve a feedback loop if *PPARD* indeed transcriptionally activates *FABP1*; however, this mechanistic link awaits experimental confirmation. This positive feedback mechanism may partially explain the molecular basis for efficient lipid deposition observed in individuals with high marbling scores.

### 4.2. Transcriptional Regulation of Lipid Droplet Formation by PPARD and Fine-Tuning of Protein Homeostasis

The formation and stabilization of lipid droplets represent the ultimate manifestation of lipid deposition [22]. For example, in human placental trophoblast cells treated with fatty acids, significant reprogramming of glycerolipid metabolism and the formation of numerous new lipid droplets can be observed [27]. PLIN2, one of the most abundant structural proteins on the lipid droplet surface, plays a critical role in stabilizing nascent lipid droplets, promoting their fusion, and protecting them from premature lipolysis [28]. This study found that *PLIN2* mRNA levels in liver tissues were increased with higher marbling scores and were positively correlated with *PPARD* expression in the cell model. Considering that the number of biological repeats in group A4 is small (*n* = 2), and the effectiveness of the statistical test is limited, this group of data is mainly used to indicate the overall trend of change; The evidence of robust expression under high-grade lipid deposition mainly came from group A3 (*n* = 10) with sufficient sample size. This correlation suggests that *PPARD* may transcriptionally influence *PLIN2* expression, potentially contributing to the initial stages of lipid droplet formation; however, this interpretation remains speculative without direct evidence of transcriptional activation.

However, in contrast to the significant changes observed at the mRNA level, PLIN2 protein levels showed no statistical differences either among marbling score groups in liver tissues or following *PPARD* interference or overexpression in cells. This phenomenon of transcription–protein decoupling is frequently reported in lipid metabolism research and may carry important physiological significance [25,29]. PLIN2 protein stability is tightly controlled by post-translational modifications. When lipid droplets form excessively or *PLIN2* is overexpressed, cells can degrade excess PLIN2 via the ubiquitin–proteasome pathway to prevent lipotoxicity and endoplasmic reticulum stress caused by excessive lipid accumulation [30,31]. In this study, although *PPARD* increased *PLIN2* transcription, the protein level remained stable, possibly due to the activation of such a negative feedback mechanism. However, as we did not experimentally validate this post-transcriptional mechanism, this interpretation remains speculative. Importantly, our data demonstrate an association between *PPARD* and *PLIN2* mRNA expression but do not establish a functional role for PPARD in lipid droplet formation, as lipid droplets or triglycerides were not directly measured. Further studies, including lipid droplet staining and triglyceride quantification, are warranted to determine whether *PPARD*-mediated changes in *PLIN2* transcription translate into functional effects on lipid droplet dynamics.

### 4.3. Hierarchical Nature of the PPARD Regulatory Network: RXR Partner Selectivity and Tissue Conservation

RXR is the universal partner of nuclear receptors [32], *PPARD* must form a heterodimer with RXR to bind to PPRE elements in the promoters of its target genes [24]. This study found that the mRNA levels of *RXRA* and *RXRB* in liver tissue significantly increased with higher marbling scores, whereas *RXRG* levels showed no differences among groups. This suggests that *RXRA* and *RXRB* are the primary functional partners of *PPARD* in bovine lipid metabolism, while *RXRG* may not be involved in this regulatory axis, possibly due to its tissue-specific distribution or differential promoter affinity [33]. The concept of non-redundant functions among different RXR subtypes has been supported in nuclear receptor research [34,35], and this study provides a new example in cattle.

Furthermore, this study performed expression analysis in liver tissue (a central organ for systemic lipid metabolism) and functional validation in bovine mammary epithelial cells (BMECs). BMECs were chosen for in vitro experiments due to their active lipid metabolism and high transfection efficiency, which enable reliable verification of *PPARD*’s regulatory effect on lipid metabolism-related genes. These cells do not simulate hepatic or skeletal muscle physiology; instead, they confirm the conserved regulatory pattern of *PPARD*, consistent with the expression trend observed in liver tissue. The direction of *PPARD*’s regulation of target genes was highly consistent across these two different sample types, suggesting that this regulatory mechanism is conserved across different lipid-metabolizing tissues in cattle. The liver, as a lipid distribution hub, may indirectly influence intramuscular fat deposition—driven by preadipocyte activation and enhanced fatty acid uptake in muscle—through *PPARD*-mediated regulation of very low-density lipoprotein (VLDL) assembly, whereas *PPARD*’s regulation of *PLIN2* in BMECs reflects its direct role in local lipid storage.

In summary, this study suggests a potential association between *PPARD* expression and the transcriptional levels of fatty acid transporter genes (*CD36*, *FATP1*) and a lipid droplet formation gene (*PLIN2*). However, as direct transcriptional regulation has not been experimentally validated, the proposed *PPARD*-mediated regulatory mechanism should be considered correlative and hypothesis-generating, requiring further functional verification. It also reveals that a positive feedback loop mediated by *FABP1* may be involved in signal amplification. Although PLIN2 protein levels are maintained homeostatically through post-translational regulation, the transcriptional initiation by PPARD remains a necessary step for lipid storage.

### 4.4. Limitations of This Study

Several limitations of this study should be acknowledged. The liver tissue samples were unevenly distributed among marbling groups, with only two animals in the A4 group, which reduces statistical power and constrains the reliability of conclusions drawn for the highest marbling category; therefore, the A4-related results should be interpreted with caution. We did not analyze muscle tissue in this study, as our focus was on hepatic lipid metabolism as a systemic contributor to marbling, given that the liver serves as a central hub for lipid distribution and may indirectly influence intramuscular fat deposition through the regulation of circulating fatty acid availability; however, direct examination of intramuscular adipose tissue would be valuable in future studies. Bovine mammary epithelial cells (BMECs) were used as an in vitro tool model due to their active lipid metabolism, conserved *PPARD* signaling across lipogenic tissues, and high transfection efficiency, which enabled reliable gene knockdown and overexpression; nevertheless, BMECs do not fully recapitulate the physiology of intramuscular adipocytes or hepatocytes, and future validation in primary intramuscular preadipocytes, mature adipocytes, or hepatocytes would be necessary to confirm the physiological relevance of our findings. Our study relied primarily on changes in gene and protein expression without functional assays directly assessing lipid accumulation, such as Oil Red O staining, triglyceride quantification, or fatty acid uptake assays. Additionally, although our results suggest that *PPARD* may be associated with the regulation of *CD36*, *FATP1*, and *PLIN2* expression, direct transcriptional regulation has not been experimentally demonstrated through ChIP-qPCR or luciferase reporter assays. Finally, while β-actin was validated as a stable reference gene across marbling groups in this study, formal reference gene validation under different experimental conditions was not performed, which represents a potential limitation. These limitations should be addressed in future investigations.

## 5. Conclusions

**Figure 12:**
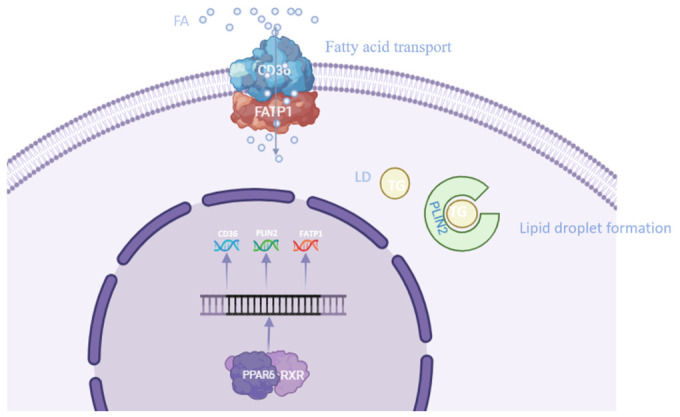
Proposed model for *PPARD*-mediated regulation of lipid deposition in cattle. *PPARD* forms a heterodimer with RXR to transcriptionally activate fatty acid transporters (*CD36*, *FATP1*) and the lipid droplet gene *PLIN2*. This pathway was validated in bovine mammary epithelial cells, representing peripheral lipogenic cells. In the liver, *PPARD*-driven lipid metabolism supports systemic fatty acid availability, which may indirectly influence intramuscular fat deposition; no direct liver-to-skeletal muscle signaling is implied.

In conclusion, this study provides evidence for an association between *PPARD* expression and the transcriptional regulation of fatty acid transport genes (*CD36*, *FATP1*, and *FABP1*) and the lipid droplet-associated gene *PLIN2* in liver tissues of cattle (Figure 12). The positive correlation between *PPARD* and these genes, supported by in vitro experiments, suggests that *PPARD* may play a role in hepatic lipid metabolism and potentially contribute to marbling development. However, our data demonstrate associations rather than mechanistic causation, and direct measurements of lipid deposition are needed. The in vitro results in bovine mammary epithelial cells support the conserved association between *PPARD* and lipid metabolism-related gene expression, offering a basis for future mechanistic studies. Overall, this study provides molecular insights into the *PPARD*-mediated pathway in bovine lipid metabolism and a foundation for further investigation of marbling formation.

## Figures and Tables

**Table 1:** siRNA sequence information.

Name	Target	Sense Strand Sequence (5′→3′)	Antisense Strand Sequence (5′→3′)
siPPARD	PPARD	GCAUGAAGCUGGAAUAUGATT	UCAUAUUCCAGCUUCAUGCTT
NC-siRNA	/	UUCUCCGAACGUGUCACGUTT	ACGUGACACGUUCGGAGAATT

**Table 2:** Primer used in the experiment.

Gene	Sequence (5′→3′)	Product Size/bp
*β-actin*	F: CTGTTAGCTGCGTTACACCCTT R: TGCTGTCACCTTCACCGTTC	166
*PPARD*	F: CGAGACAGCCTTGTGTGGTGTT R: GCAGTTCCCGTCAGCCTCTTTG	108
*FATP1*	F: GAGCCTGGTCAAGTTCTGTTCTG R: GTAGGAGTAGTGCCCAAATGCC	238
*FABP1*	F: TCCAGACCCAGGAGAACTATGAG R: ATCACCTTCCTGCTGAACCACT	239
*PLIN2*	F: CTTGCTATTGCCCGGAACC R: CCTAAAGCCACAGGAATGAACAC	498
*CD36*	F: GTACAGATGCAGCCTCATTTCC R: TGGACCTGCAAATATCAGAGGA	87
*RXRA*	F: TTCTCCACGCAGGTGAACTC R: CGTAGTGCTTGCCTGAGGAG	427
*RXRB*	F: GGGCTGGCAAACGGCTAT R: TGTTGTCCCGGCACGAGTA	138
*RXRG*	F: CTTGTCGGGATAACAAAGACTGC R: TGACTTGGTCCTCCAAGGTGA	378

**Table 3:** Reaction reagent.

Reagent	Dosage/μL
SYBR Green I qPCR Mix	10
upstream primer	0.5
downstream primer	0.5
cDNA	1
DEPC water	8
total volume	20

## Data Availability

The original contributions presented in this study are included in the article. Further inquiries can be directed to the corresponding authors.

## Supplementary Materials

The following supporting information can be downloaded at: https://www.mdpi.com/article/10.3390/ani16132096/s1, Figure S1: Western blot analysis of PPARD protein expression in bovine liver samples with different marbling grades; Figure S2: Western blot analysis of β-actin as a loading control in bovine liver samples with different marbling grades; Figure S3: Western blot analysis of PLIN2 protein expression in bovine liver samples with different marbling grades; Figure S4: GAPDH expression in bovine mammary epithelial cells under different transfection conditions; Figure S5: PPARD expression in transfected bovine mammary epithelial cells; Figure S6: PLIN2 expression in transfected bovine mammary epithelial cells; Figure S7: β-actin expression in bovine mammary epithelial cells under different transfection conditions.
