# Supplementary material for: Differences of PPARD Expression in the Liver of Cattle with Different Marbling Grades
Source: Animals (Basel). 2026 Jul 6;16(13):2096. doi: 10.3390/ani16132096 (PMC13359835; doi:10.3390/ani16132096)
Supplement: Supplementary file 1 [file animals-16-02096-s001.zip › animals-4393245-supplementary.pdf]

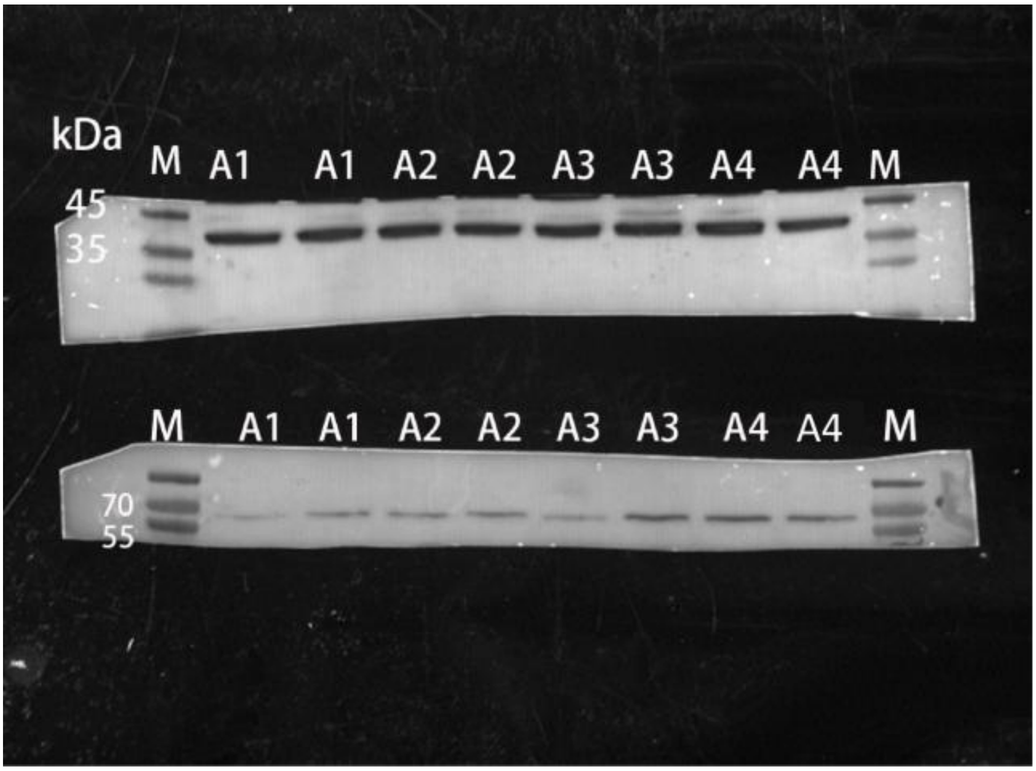

Supplementary Figure S1. Western blot analysis of PPARD protein expression in bovine liver samples with different marbling grades.

The expression pattern of PPARD was determined in bovine liver tissues with four marbling grades (Grade 1 to Grade 4, corresponding to A1–A4). The upper bands represent GAPDH at 36 kDa as the internal reference, and the lower bands show PPARD protein at 56 kDa. Each group was set with biological replicates. The uniform gray level of GAPDH bands indicated consistent protein loading among all samples. Specific and distinct PPARD bands were observed in each group, which laid a foundation for the subsequent quantitative analysis of PPARD expression in bovine liver with different marbling levels.

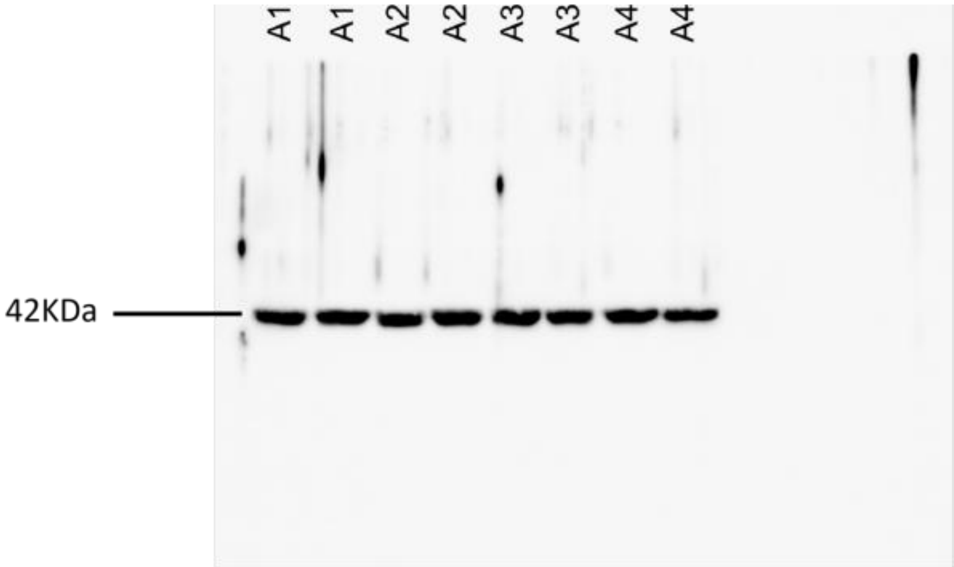

Supplementary Figure S2. Western blot analysis of β-actin as a loading control in bovine liver samples with different marbling grades.

The expression pattern of β-actin was determined in bovine liver tissues with four marbling grades (Grade 1 to Grade 4, corresponding to A1–A4). β-actin protein was detected at approximately 42 kDa as the internal reference. Each group was set with biological replicates. The uniform intensity of β-actin bands indicated consistent protein loading among all samples, which laid a foundation for the subsequent quantitative analysis of target protein expression in bovine liver with different marbling levels.

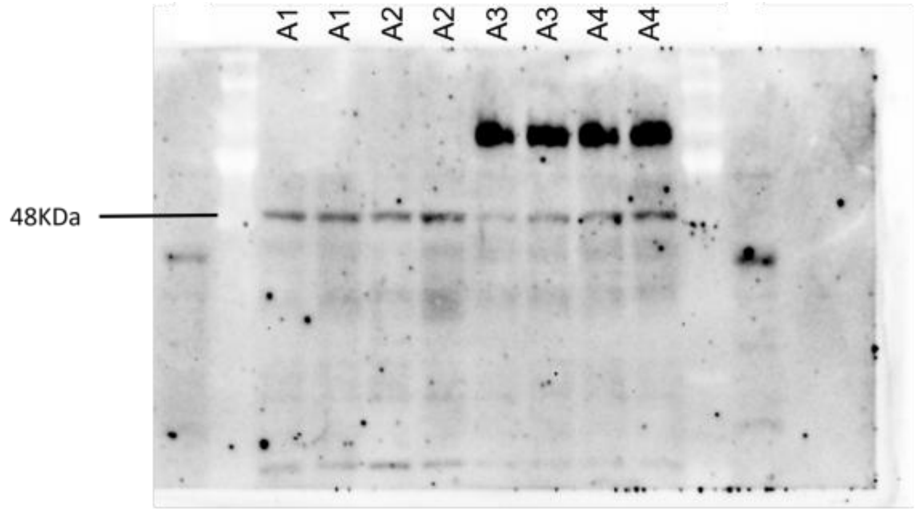

Supplementary Figure S3. Western blot analysis of PLIN2 protein expression in bovine liver samples with different marbling grades.

The expression pattern of PLIN2 was determined in bovine liver tissues with four marbling grades (Grade 1 to Grade 4, corresponding to A1–A4). PLIN2 protein was detected at approximately 48 kDa. Each group was set with biological replicates. Specific and distinct PLIN2 bands were observed in each group, which laid a foundation for the subsequent quantitative analysis of PLIN2 expression in bovine liver with different marbling levels.

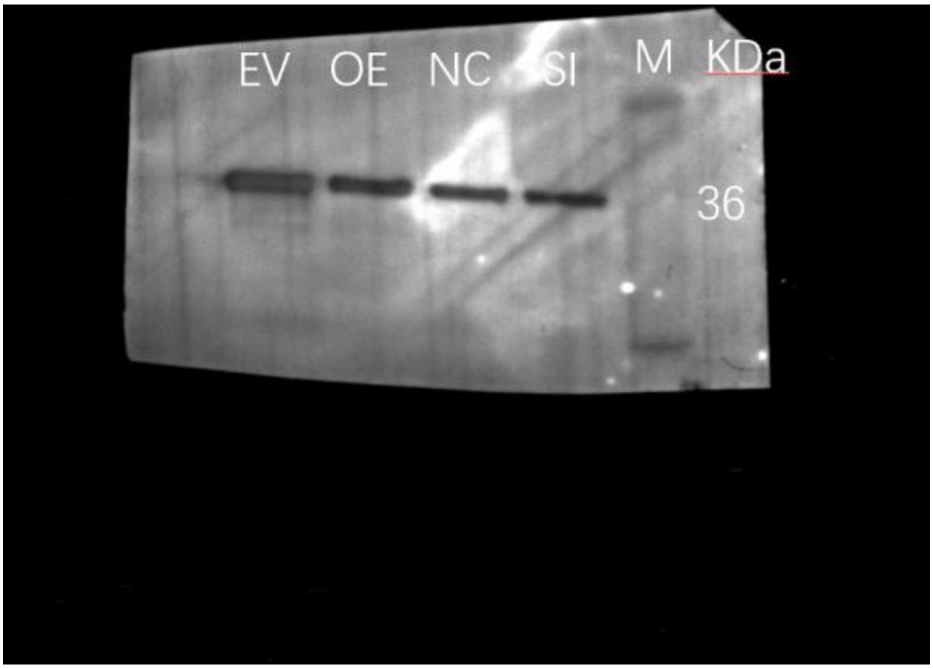

Supplementary Figure S4. GAPDH expression in bovine mammary epithelial cells under different transfection conditions.

Western blot analysis of the housekeeping protein GAPDH was performed in bovine mammary epithelial cells subjected to different genetic manipulations: empty vector control (EV), PPARD overexpression (OE), negative control siRNA (NC), and PPARD knockdown siRNA (SI). GAPDH was detected at its expected molecular weight of approximately 36 kDa. The consistent band intensity across all four groups confirmed equal protein loading, validating the reliability of the internal control for subsequent target protein normalization.

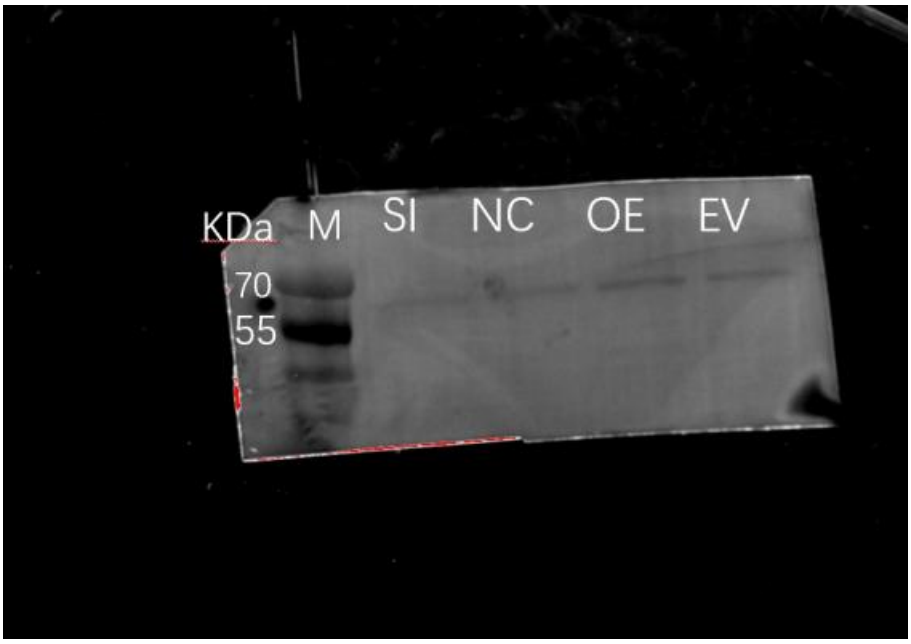

Supplementary Figure S5. PPARD expression in transfected bovine mammary epithelial cells.

Western blot was used to assess PPARD protein levels in bovine mammary epithelial cells after transfection with PPARD siRNA (SI), negative control siRNA (NC), PPARD overexpression vector (OE), or empty vector (EV). A single band corresponding to PPARD was detected at ~56 kDa. The band intensity was increased in the OE group and reduced in the SI group relative to the NC and EV controls, confirming the effectiveness of the transfection

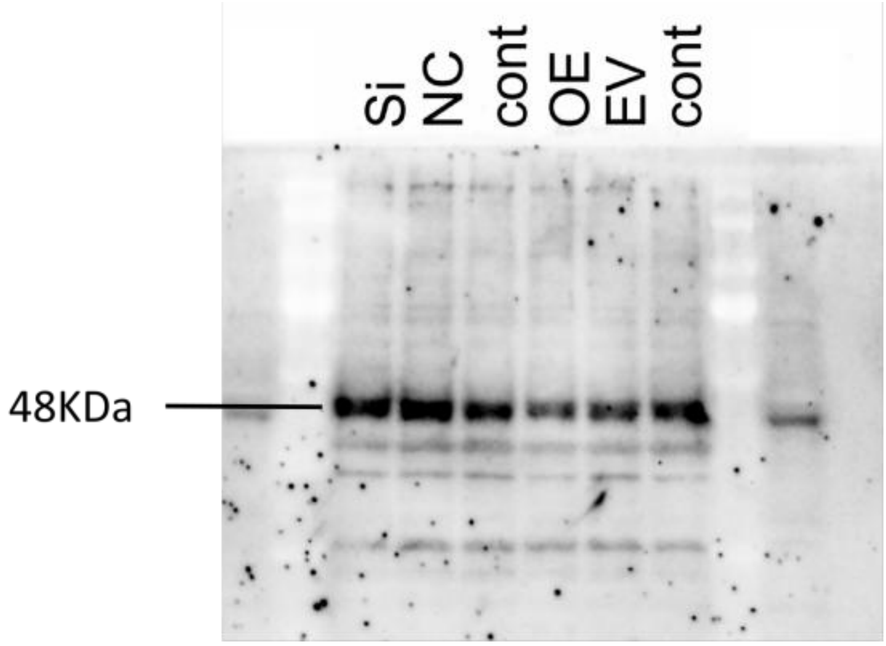

Supplementary Figure S6. PLIN2 expression in transfected bovine mammary epithelial cells.

Western blot was used to assess PLIN2 protein levels in bovine mammary epithelial cells after transfection with PPARD siRNA (SI), negative control siRNA (NC), PPARD overexpression vector (OE), or empty vector (EV). A single band corresponding to PLIN2 was detected at ~48 kDa. The band intensity varied across groups relative to the controls, indicating that PPARD manipulation affects PLIN2 protein expression in these cells.

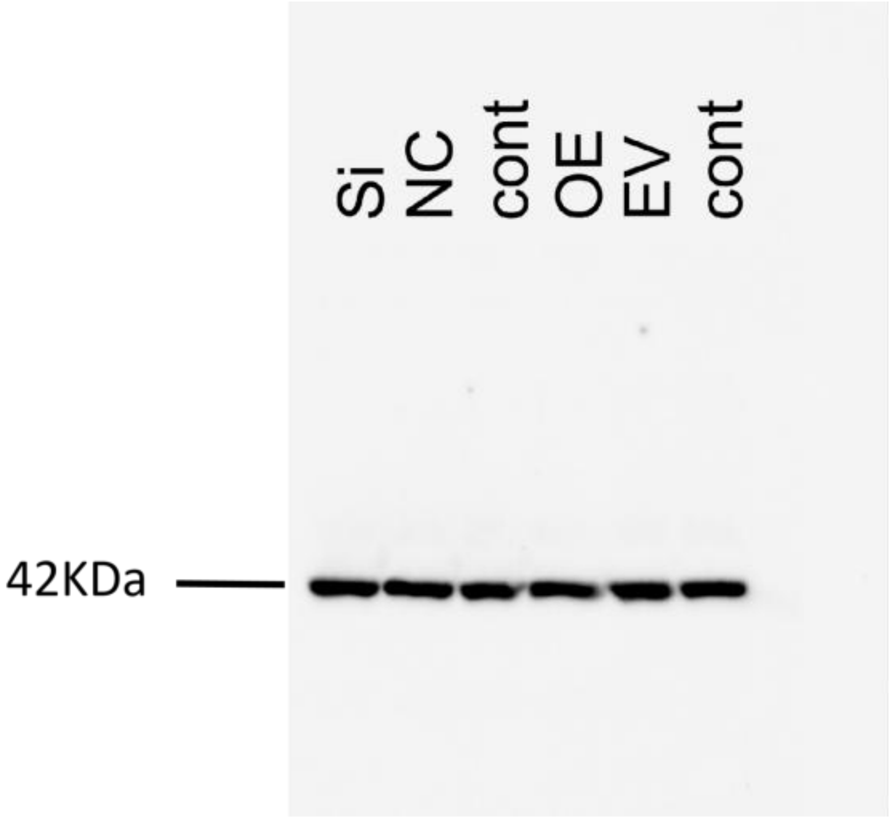

Supplementary Figure S7. β-actin expression in bovine mammary epithelial cells under different transfection conditions.

Western blot analysis of the housekeeping protein β-actin was performed in bovine mammary epithelial cells subjected to different genetic manipulations: empty vector control (EV), PPARD overexpression (OE), negative control siRNA (NC), and PPARD knockdown siRNA (SI). β-actin was detected at its expected molecular weight of approximately 42 kDa. The consistent band intensity across all four groups confirmed equal protein loading, validating the reliability of the internal control for subsequent target protein normalization.
